# Triticale Green Plant Regeneration Is Due to DNA Methylation and Sequence Changes Affecting Distinct Sequence Contexts in the Presence of Copper Ions in Induction Medium

**DOI:** 10.3390/cells11010084

**Published:** 2021-12-28

**Authors:** Renata Orłowska, Katarzyna Anna Pachota, Piotr Androsiuk, Piotr Tomasz Bednarek

**Affiliations:** 1 Department of Plant Physiology and Biochemistry, Plant Breeding and Acclimatization Institute–National Research Institute, 05-870 Błonie, Poland; katarzyna.anna.pachota@gmail.com (K.A.P.); p.bednarek@ihar.edu.pl (P.T.B.); 2 Department of Plant Physiology, Genetics and Biotechnology, Faculty of Biology and Biotechnology, University of Warmia and Mazury in Olsztyn, 10-719 Olsztyn, Poland; piotr.androsiuk@uwm.edu.pl

**Keywords:** androgenesis, copper, genetic variation, metAFLP, silver, symmetrical/asymmetrical methylation context, time, triticale

## Abstract

Metal ions in the induction medium are essential ingredients allowing green plant regeneration. For instance, Cu(II) and Ag(I) ions may affect the mitochondrial electron transport chain, influencing the Yang cycle and synthesis of S-adenosyl-L-methionine, the prominent donor of the methylation group for all cellular compounds, including cytosines. If the ion concentrations are not balanced, they can interfere with the proper flow of electrons in the respiratory chain and ATP production. Under oxidative stress, methylated cytosines might be subjected to mutations impacting green plant regeneration efficiency. Varying Cu(II) and Ag(I) concentrations in the induction medium and time of anther culture, nine trials of anther culture-derived regenerants of triticale were derived. The methylation-sensitive AFLP approach quantitative characteristics of tissue culture-induced variation, including sequence variation, DNA demethylation, and DNA de novo methylation for all symmetric-CG, CHG, and asymmetric-CHH sequence contexts, were evaluated for all trials. In addition, the implementation of mediation analysis allowed evaluating relationships between factors influencing green plant regeneration efficiency. It was demonstrated that Cu(II) ions mediated relationships between: (1) de novo methylation in the CHH context and sequence variation in the CHH, (2) sequence variation in CHH and green plant regeneration efficiency, (3) de novo methylation in CHH sequences and green plant regeneration, (4) between sequence variation in the CHG context, and green plant regeneration efficiency. Cu(II) ions were not a mediator between de novo methylation in the CG context and green plant regeneration. The latter relationship was mediated by sequence variation in the CG context. On the other hand, we failed to identify any mediating action of Ag(I) ions or the moderating role of time. Furthermore, demethylation in any sequence context seems not to participate in any relationships leading to green plant regeneration, sequence variation, and the involvement of Cu(II) or Ag(I) as mediators.

## 1. Introduction

Metal ions are ingredients commonly added to the induction medium (IM) of cereals’ anther culture [1,2]. The maintenance of metal homeostasis is vital for the functioning of mitochondria. At high concentrations, they might be toxic to the cell, affecting for example, functional sites of proteins or enhancing reactive oxygen species (ROS) production [3]. The ROS originate either from chloroplasts or mitochondria [4,5]. In mitochondria, ROS are generated as the result of the electron transport chain (ETC) functioning. The ETC of plant mitochondria contains complexes I–IV/V that, among others, encompass many enzymes (an alternative oxidase (AOX) [6] and four NAD(P)H dehydrogenases) [7]). Copper (I) and (II) redox properties are used by cytochrome *c* oxidase (COX), the terminal enzyme of the ETC. Its functioning requires the action of multiple factors that are involved in the delivery and insertion of copper into the enzyme [8]. Copper and iron, as redox metals, directly induce ROS creation through Fenton and Haber–Weiss reactions [9]. Furthermore, copper ions form complexes with metalloproteins and can be bound by L-histidine-Cu(II) [10], L-cysteine-Cu(I) [11] and methionine-Cu(I) [12]. Thus, disturbances in the ETC and impaired ATP production may influence the Yang cycle and synthesis of the S-adenosyl-L-methionine (SAM) metabolite [13], which is the universal methyl-group donor in the cell [14,15,16]. Furthermore, cysteine synthesis relies on methionine and is a rate-limiting precursor of glutathione (GSH) synthesis. GSH is a small intracellular thiol and a strong non-enzymatic antioxidant [17]. It is also the final product of the transsulfuration pathway [18]. The disturbances in S-adenosyl-L-methionine synthesis may result in changes in the DNA methylation pattern [19]. DNA methylation changes are critical in anther cultures, as a switch from gametophytic fate into sporophytic one takes place during the cell reprogramming stage [20]. Primary DNA demethylation during cell dedifferentiation needs to be reestablished via de novo methylation [20]. The process involves both genetic and epigenetic mechanisms [21]. It is worth noting that DNA methylation in plants occurs in three contexts: CG, CHG, and CHH (where H is any base except G). Moreover, each of these contexts is regulated differently by four DNA methyltransferase (DNMT) families. Methylated cytosine residues are sensitive towards oxidative stress [22] possibly leading towards mutations [23,24]. It was demonstrated that DNA methylation changes related to copper ions present in the IM might influence sequence variation and green plant regeneration efficiency (GP) in barley [25,26]. Furthermore, silver ions added to the IM might also contribute to the GP regeneration [27]. It has being suggested that silver ions have a similar size as copper, and thus, they can substitute copper in its complexes [28]. However, copper ions seem to be of priority in barley zygotic embryo cultures [25], with no apparent changes in plant morphology. In contrast, in barley anther cultures, Ag(I) and Cu(II) ions influenced the GP regeneration [27] indicating different aspects of biochemical pathways functioning in the two regeneration approaches. Finally, it is being proposed that the time of in vitro anther culture may influence tissue culture-induced variation (TCIV) and the GP regeneration. The putative role of the time was demonstrated in anther culture of pepper [29], turnip rape [30] and barley [27], but was not documented in triticale. However, in some instances, the role of time is being questioned [31].

We hypothesize that copper and silver ions in the IM of triticale, anther culture similar to barley anther cultures, may affect the electron transport chain as the final stage of respiration and the Yang cycle. Disturbances of the Yang cycle are followed by DNA methylation changes (including symmetric and asymmetric sequence contexts). The changes may be subjected to genomic sequence mutations influencing the GP, and that the phenomenon could be sensed using the methylation-sensitive AFLP (metAFLP) approach developed for the quantification of TCIV [32,33,34,35]. Furthermore, we want to verify the role of the time of triticale anther culture for the TCIV. Also, the relationships between analyzed variables could be illustrated via the regression-based mediation analysis method [36] to evaluate complex relationships between many variables reflecting the cellular phenomena [37].

## 2. Materials and Methods

### 2.1. Tissue Cultures

**Figure 1:**
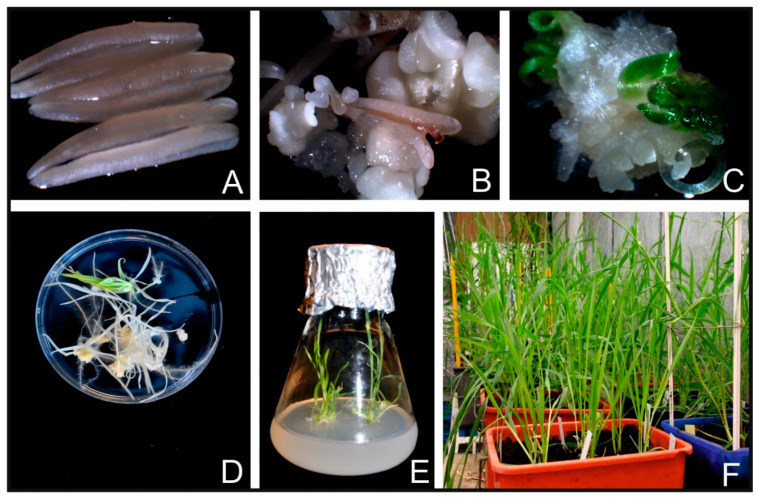
Schematic representation of plant regeneration via in vitro culture. (**A**) anthers on solid medium; (**B**) embryo formation from anther culture; (**C**) embryos and germinating embryos; (**D**) albino and green plants derived from anther culture, (**E**) green regenerants transferred to glass flask; (**F**) green developing regenerants in pots.

Progeny of doubled haploid winter triticale (x *Triticosecale* spp. Wittmack ex. A Camus 1972) cultivar T28/2 derived from cv. Mungis x cv. Presto provided by Sylwia Oleszczuk (Plant Breeding and Acclimatization Institute-National Research Institute, Radzików, Poland) was used as donor plants to obtain regenerants via androgenesis in anther culture. Twenty-four triticale donor plants were cultivated in controlled conditions in a growth chamber. The plants grew at a temperature of 16 °C during the day and 12 °C at night. The photoperiod was: 16 h day and 8 h night. Vernalization was carried out at 4 °C for six weeks in a photoperiod of 8 h day and 16 h night. nine, the plants were grown in a greenhouse under a photoperiod (16 h/8 h day/night). Light conditions were maintained using high pressure sodium lamps between 6:00 a.m.–8:00 a.m. and 6:00 p.m.–10:00 p.m., natural light was used during the remaining hours. Plants were watered twice a week. Tillers of donor plants were harvested when microspores were mid- to the uninucleate stage. To induce androgenesis, the tillers were subjected to cold stress (4 °C) in the dark for 20 days. The surface-sterilization of spikes was carried out by soaking in 70% ethanol for 1 min, followed by soaking in 10% sodium hypochlorite for 20 min and washing four times with sterile deionized water. Then, anthers (Figure 1A) were removed from spikes and laid on solid induction media in the laminar flow cabinet. As an induction medium was used (190-2 medium [38] with 90 g L^−1^ maltose) (Duchefa Biochemie, Haarlem, The Netherlands) and 438 mg L^−1^ glutamine (Duchefa Biochemie, Haarlem, The Netherlands) supplemented with 2 mg L^−1^ 2,4-dichlorophenoxyacetic acid (2,4-D) (Duchefa Biochemie, Haarlem, The Netherlands) and 0.5 mg L^−1^ kinetin (Duchefa Biochemie, Haarlem, The Netherlands). Induction media were supplemented with CuSO_4_ (Sigma Aldrich, Steinheim, Germany) (0.1, 5, 10 µM) and AgNO_3_ (Sigma Aldrich, Steinheim, Germany) (0, 10, 60 µM) salts (Table 1), that nine trials were prepared. 0.1 µM Cu (II) is a standard concentration of copper commonly used in cultivation media. Each medium trial was assigned a different time of anthers incubation (35, 42, 49 days), including time from plating explants on induction media to calli collection and transferred them on regeneration media. Androgenic structures (callus, embryo-like structures, and embryos) (Figure 1B,C) were transferred successively on a regeneration medium starting from the 35 days of culture in one-week intervals. Here was used regeneration medium 190-2 [38] supplemented with 0.5 mg L^−1^ naphthalene acetic acid (NAA) (Sigma Aldrich, Steinheim, Germany) and 1.5 mg L^−1^ kinetin.

After six weeks of regeneration, green plantlets (Figure 1D) were transferred to glass flasks (Figure 1E) with N6I medium [39] supplemented with 2 mg L^−1^ indole-3-acetic acid (IAA) (Duchefa Biochemie, Haarlem, The Netherlands) for rooting. Later, developed green plants (Figure 1F) were potted into containers filled with soil and sand and grown in a greenhouse. Next, seedlings were vernalized at 4 °C for six weeks and were shifted to the greenhouse and grown under controlled conditions until harvest. The number of green plants (GPs) was assessed as the number of green regenerants obtained per 100 plated anthers. In addition, phenotypic data concerning plants’ height, leaves’ shape, leaves’ color and ability to set seeds were inspected visually. The donor plants and the regenerants obtained from them were visually assessed. 

### 2.2. DNA Extraction and metAFLP Assay

Genomic DNA was extracted from young leaves (tillering stage) of regenerants and donor plants according to the Plant DNeasy MiniPrep Kit (Qiagen, Hilding, Germany). For each metAFLP reaction, there was prepared 500 ng of DNA of spectrophotometrically verified quantity and purity. The metAFLP procedure was conducted according to previously developed protocols [32,40]. DNA from each regenerant was divided into two portions of 500 ng each and cut in parallel with two sets of restriction enzymes *Acc65*I/*Ms*eI and *Kpn*I/*Mse*I (New England Biolabs, Ipswich, MA, USA). The obtained DNA fragments were ligated with synthetic oligonucleotides (adapters) in a ligation reaction followed by an initial PCR reaction with preselective primers. The product of the preselective PCR reaction was used as a template for the selective PCR with selective primers (Supplementary Table S1), where one primer was radioactively labeled (P^32^, Hartmann Analytic GmbH, Braunschweig, Germany). The products of selective PCR were separated in a 7% polyacrylamide gel. Visualization of fractionated DNA fragments was performed by exposing the gels to X-ray film (Fujifilm Corporation, Tokyo, Japan).

### 2.3. Quantifying Variation

Estimation of TCIV was based on the presence (1) or absence (0) of bands (DNA fragments) for donors and regenerants on a polyacrylamide gel. Binary matrices were created based on the compilation of DNA profiles obtained from *Acc65*I/*Mse*I and *Kpn*I/*Mse*I enzyme cuts. Based on the properties of the restriction enzymes used, it was possible to determine the changes in the DNA of regenerants caused by in vitro culture. Sequence variation (SV), demethylation (DMV), and de novo methylation (DNMV) were determined. Selective primers terminated with any combination of A and T could amplify the asymmetric CHH sequence. MetAFLP primers terminated with the -CHG sequence at the 3′-end and those with the -CG sequence reflect symmetric contexts. Thus, selective primers targeting symmetric and asymmetric DNA sequences used in the metAFLP technique enabled identification of CG, CHG, and CHH methylation change contexts as well as sequence changes related to those sequences. The details of quantifying changes in different sequence contexts concerning DNMV, DMV and SV were described elsewhere [33].

### 2.4. Statistics

Simple mediation analyses were conducted in IBM SPSS software v 27 (IBM Corop, NY, USA) using PROCESS Macro 3.5 [36]. The statistical power of hierarchical linear regression and simple moderation was evaluated using G-Power software [41]. The Lasso regression analysis was conducted in XLSTAT software (Addinsoft, NY, USA) [42] using implemented GLMNET R package and predefined settings. Linear multiple regression using *F* distribution and fixed model, *R*^2^ deviation from zero with *post hoc* power analysis was employed.

## 3. Results

Nine different treatments (trials M1-M9, Table 1) varying in copper and silver ion concentrations in IM and time of cultures resulted in 44 regenerants. All regenerants were evaluated for morphological characteristics and then used in molecular analyses. Plants with set seeds, and thus fertile plants, were scored as those with doubled chromosome numbers. These regenerants determine the success of the procedure to obtain doubled haploid (DH) plants. Depending on the trial, the number of regenerated green DH plants varied from 3 to 10. All regenerants were in a type of donor plant without any morphological differences concerning leaves’ shape, color, plants’ height, time of flowering, and seed set.

DNA extraction from young leaves of donors and regenerants (collected at the same developmental stage-tillering) resulted in integral and intact samples. After digestions with the *Acc65*I/*Mse*I and *Kpn*I/*Mse*I endonucleases following the metAFLP procedure, DNA samples amplified 574 and 555 easily scorable repeatable banding profiles for each of the metAFLP platforms. In addition, 295 and 266 markers were polymorphic. The metAFLP profiles, including the presence or absence of a given band in donor and regenerant in the *Acc65*I/*Mse*I and *Kpn*I/*Mse*I metAFLP platforms, were classified into sixteen patterns reflecting different event types (sequence change -SV, de novo methylation-DNMV, DNA demethylation-DMV). The events allowed the evaluation of quantitative characteristics of sequence variation, de novo DNA methylation, and DNA demethylation variation, including symmetric (CG, CHG) and asymmetric (CHH) sequence contexts (Table 1).

**Figure 2:**
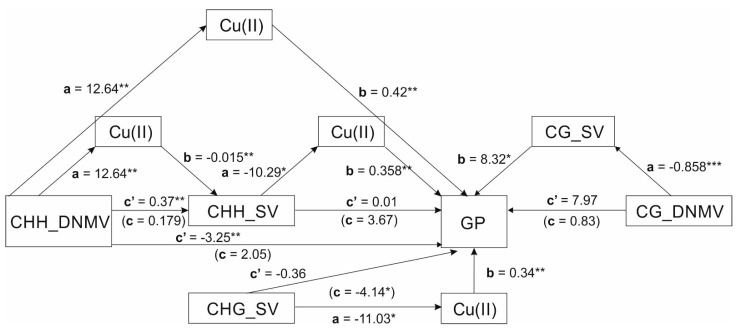
Schematic representation of all mediation analysis in a combined form. The unstandardized coefficients of paths a, b, c’, and c (in brackets) are indicated on respective arrows. CHH_DNMV, CHH_DNMV, CHG_DNMV, CG_DNMV, and CG_SV are the metAFLP quantitative characteristics in CHH, CHG and CG sequences; Cu(II) states for copper ions whereas GP for green plants derived via anther culture of triticale. *, **, ***—significance at *p* = 0.05, 0.001, or 0.0001, respectively.

A series of regression mediation analyses (Figure 2) showed that copper ions affecting distinct contexts of de novo methylation mediated relationships between methylation contexts and sequence variation were evaluated.

A regression mediation analysis (Table 2) showed that copper ions affecting de novo methylation resulted in sequence variation (DNMV → Cu → SV). Detailed analysis of the relationship showed that the identified mediation was due to the relationship between de novo methylation in CHH context and sequence variation in CHH one mediated by Cu(II) (CHH_DNMV → Cu → CHH_SV). Moreover, de novo methylation in the CHH context mediated by copper ions affected the number of green regenerants obtained per 100 regenerants (CHH_DNMV → Cu → GP). Furthermore, copper ions also mediated the relationship between sequence variation in CHH context (and sequence variation in CHG context) and GPs (CHH_SV → Cu → GP and CHG_SV → Cu → GP). Finally, the relationship between de novo methylation in CG context and the number of GPs was mediated by sequence variation in CG context (CG_DNM → CG_SV → GP).

The significance of mediations was confirmed by bootstrap confidence intervals of indirect effects that did not contain zero. No mediations involving silver ions as mediators between SV and DNMV (or DMV) in all sequence contexts or GP were found. Similarly, DMV (all contexts) did not participate in mediation analyses either as predictors or mediators of SV or GPs. The relationships between de novo methylation in CHH context (CHH_DNMV) and GP were mediated by copper ions, whereas de novo methylation in CG context (CG_DNMV) and GP by sequence variation in CG one (CG_SV). Furthermore, the time of tissue culture failed to be a moderator in between independent variables (DNMV, CHH_DNMV, CHG_DNMV) and copper ions in all respective mediation analyses (Table 2). Therefore, only significant mediation results are listed in Table 2.

Lasso regression used to select most influencing predictors (Table 1) used in mediation analyses showed that coefficients not equal to zero and corresponding to optimal lambda (0.0028) resulted in the following equation: GP =-51.11 + 7.22 × CG_SV-5.065 × CHH_DNMV + 3.04 × CG_DNMV + 2.81 × CHH_SV − 2.43 × CHG_SV + 0.36 × Cu(II). Thus, all predictors were significant for predicting GPs. Sequence variation affecting CG context increased, whereas the CHG decreased the GPs. The influence of DNMV was negative for the CHH context but positive for the CG, whereas the role of copper ion concentration on GPs seems to be the smallest.

Assuming the total sample size equated to 44, α = 0.05, and medium effect size, the statistical power of mediation was 0.82.

## 4. Discussion

It is being suggested that copper ions are required by Cu/Zn dependent superoxide dismutase (SOD1) and for cytochrome *c* oxidase in the respiratory chain, and that indirectly affects the Yang cycle [43]. The ion concentration disturbances may result in DNA methylation level changes [19,44]. Moreover, copper ions may participate in reactions with methylated cytosine residues leading to SV [25]. Our results demonstrate that copper ions mediated relationships between CHH_DNMV and CHH_SV that may reflect the role of copper as a cofactor in biochemical pathways [45] affecting epigenetic mechanisms [46]. Furthermore, copper ions also mediated relationships between SV influencing CHH sequence contexts and GPs (CHH_SV → Cu → GP). The same relationship was shown for SV of the CHG contexts and GPs (CHG_SV → Cu → GP). Sequence variation reflecting CHH context participates in the number of green plants regenerated via anther culture involving copper as a mediator (CHH_SV → Cu → GP). The process might be initiated via de novo methylation of CHH sequences. The evaluation of de novo methylation of the CHH asymmetric sequence (which is under epigenetic control [47]) and the de novo methylation of symmetric CG sequences (controlled by genetic mechanisms during DNA replication [47,48] and is subjected to the cellular repair machinery [49]) without copper ions participation suggests, that both epigenetic and genetic mechanisms might be involved in GP regeneration via anther culture in triticale.

It is well-known that methylation of the CHG sequence context is under genetic and epigenetic mechanism control. Our result may suggest that epigenetic aspects may be less pronounced than genetic ones in the case of GP regeneration and/or the number of regenerants (sample size) evaluated in our experiment is too small to detect the input of copper ions in this case. In general, the presented results are in agreement with those for barley regenerants derived from zygotic embryos [25] where it was shown that de novo methylation was influenced by copper ions. Furthermore, time of culture acted as a moderator affecting the yield of GPs [27]. It should be stressed, however, that in the GPs of barley time was significant not only in de novo methylation but also in DNA demethylation [27] which seems to be not the case in triticale. Interestingly, analyses of global DNA methylation changes in both species showed that in triticale [50] and barley [51], the direction of changes is different. In triticale, a lowered level of global methylation in regenerants compared to donors was shown, suggesting genome demethylation. However, in barley, the direction is opposite, and there is a higher level of global DNA methylation in regenerants. Furthermore, in barley the role of Ag(I) used as ingredients for in vitro culture medium in the production of GP or its relationships with sequence variation or DNA methylation change (DNMV, DMV in CG and CHG contexts) was demonstrated [44] but was not shown in triticale anther culture-derived regenerants. The lack of identified relationships of Ag(I) with DMV, DNMV, or GPs is surprising as silver ions are supposed to substitute copper in SOD complexes [52,53] affecting its proper functioning and leading towards sequence variation [54] or enhancing the regeneration processes in oat anthers [2]. We tend to think that the involvement of Ag(I) in varying relationships in triticale is masked by limited sample size, or its role is less pronounced in comparison to barley. The notion seems to be confirmed by the fact that in triticale, we observed increased demethylation of the regenerants’ genome [50], whereas in barley, the opposite was observed [51]. Additional experiments are needed to understand the differences between GPs in the two species. The other discrepancy between barley and triticale regenerants is related to the longevity of tissue culture. It is widely accepted that time of in vitro culture positively influences SV [55]. Still, in triticale, the relationships between SV, DNMV, DMV in any sequence context and Cu(II) and time of anther culture were not revealed. The reason for that is not apparent, and both statistical issues and biological phenomena should be considered.

The relationships discussed above implicated that regeneration of green plants in triticale is a complex phenomenon affecting DNMV and SV in different sequence contexts and that copper ions may mediate relationships between them. It is not evident which of the players have the most significant input into GPs. However, applying Lasso regression analysis, one may try to identify such factors. However, the analysis showed that all predictors of GP were influential. The CG_SV, in contrast to CHG_SV and CHH_SV, positively influenced the GP values. This could be related to the genetic mechanism of DNA methylation pattern reestablishment of the symmetric CG contexts [47,48,49] in contrast to the CHH [56] and partially CHG contexts [57,58]. If the CHH and CHG sequence contexts are methylated and then modified cytosine residues are subject to mutations, then the repairing of such sequences (especially CHH) is not so precise as such contexts are either completely (CHH) or partly (CHG) controlled by epigenetic mechanisms. The notion is also supported by a positive coefficient of CG_DNMV and a negative one for CHH_DNMV. Furthermore, the lasso regression results have demonstrated that most predictors influencing GP mediated by copper ions (CHH_DNMV, CHG_SV) negatively influence green plant regeneration, whereas CG_SV and CG_DNMV variations not relaying on Cu(II) as a mediator positively affect the GP. Furthermore, Cu(II) also positively influences GP regeneration. In the animal genomes CHH methylated sequences may undergo mutations following oxidative mechanism that results in 5-formylcytosine (5fC), 5-carboxycytosine 5caC), and 5-hydroxymethylcytsine (5hmC). The first two modifications are rapidly repaired by the base excision mechanism [59] and are involved in the active DNA demethylation pathway [60]. The third modification is stable under physiological conditions and likely has implications on many biological processes [61]. It cannot be excluded that the same may be true for the plant genome, where 5caC, 5fC, and 5mhC have also been identified [62]. And the processes themselves concerning the active or passive removal of the 5hmC form are still under investigation [63,64]. Besides, the positive action of the CG_DNMV and CG_SV on the GP regeneration could be explained by the fact that in seed plants, CG sequences’ methylation is low at the edges of genes and high in the gene body [65,66,67,68]. Thus, sequence mutations affecting CG genomic regions due to cytosine residues’ de novo methylation increasing heterozygosity influence the GPs regeneration as indicated by the positive effect of the CG_SV and CG_DNMV. On the other hand, the negative role of the CHH_DNMV may be explained by the fact that such sequences are highly methylated in the proximity of genes [65,66,67,68] possibly influencing gene regulatory regions. Interestingly, sequence mutations of the CHH contexts related to cytosine methylation positively affect the GPs, which might be explained by either increased heterozygosity of the DH plants or the increased availability of the regulatory sequence putative gene expression. Remarkably, sequence mutations of the CHG sequences, usually being low methylated in the gene body [65,66,67,68] also could be expected to increase the DH plants’ heterozygosity and the GP number, but this is not the case. It is possible that mutations in the CHG sequences may result in C→T transitions, resulting in a stop codon (UAG) [69] and, thus, gene expression abortion. Unfortunately, we failed to implement the CHG_DNMV as a mediator. Thus, we have no data to support the notion that cytosine deamination in the CHG contexts is an essential factor here.

The limitation of the study could be the sample size which might affect statistical power. However, the evaluation of green plants under varying in vitro tissue culture conditions may be problematic, as was the case here, in triticale. Still, we have succeeded in regenerating 44 plants sufficient to achieve 0.82 of statistical power for all analyses. Thus, moderation analysis was assumed to be conclusive, illustrating putative relationships between analyzed variables.

## 5. Conclusions

The presented data demonstrates that triticale green plant regeneration via anther culture seems to be influenced by sequence variation, including distinct sequence contexts. In some cases, de novo methylation of the sequences precedes SV. Interestingly CHH and CHG contexts affecting GP are controlled by copper ions in contrast to the CG sequences. Thus, it seems that distinct sequence contexts have a different effect on GPs. Furthermore, CG_SV seems to be influential predictor of GPs. The mediation model presented here assumes that the addition of Cu(II) to IM affects cytosine methylation within CHH contexts, resulting in SV. In turn, sequence variation and de novo methylation within CHH contexts lead to increased regeneration efficiency of green regenerants. This last observation is essential from a practical point of view because androgenesis in some cereal plants is a process with low efficiency.

Moreover, the models, which describe the relationships between the components of the culture media, concerning biochemical cycles and the efficiency of androgenesis may have their practical aspect leading not only to a better understanding of plant regeneration but also to the possibility of improving the efficiency of obtaining plants that are doubled haploids.

The involvement of Cu(II) in the GPs suggests that disturbances of Cu(II) ions present in the IM may affect the ETC in the mitochondrial respiration chain and/or the Yang cycles, influencing the de novo DNA methylation required for sequence variation in the case of some sequence contexts. Still, analysis involving an enlarged sample size is needed to reveal more complex relationships between analyzed characteristics reflecting cellular phenomena.

## Figures and Tables

**Table 1:** Quantitative metAFLP characteristics and respective tissue culture conditions used for DH plant regeneration by trials (M1–M9).

No.	Trial	metAFLP Characteristics ^1^ (%)												In Vitro Conditions			GP
		SV	DNMV	DMV	Sequence CHH			Sequence CHG			Sequence CG						
					SV	DNMV	DMV	SV	DNMV	DMV	SV	DNMV	DMV	Cu(II) (μM)	Ag(I) (μM)	Time (Days)	
1	M1	43.30	3.34	5.02	8.66	0.37	0.74	23.34	1.62	2.92	11.35	1.33	1.33	0.1	0	35	0.91
2	M1	43.62	3.18	5.01	8.66	0.37	0.74	23.49	1.62	2.92	11.51	1.17	1.33	0.1	0	35	0.91
3	M1	43.61	3.18	5.18	8.66	0.37	0.74	23.49	1.62	2.92	11.51	1.17	1.50	0.1	0	35	0.91
4	M1	43.61	3.18	5.18	8.66	0.37	0.74	23.49	1.62	2.92	11.51	1.17	1.50	0.1	0	35	0.91
5	M1	43.62	3.18	5.01	8.66	0.37	0.74	23.49	1.62	2.92	11.51	1.17	1.33	0.1	0	35	0.91
6	M2	43.61	3.18	5.18	8.66	0.37	0.74	23.49	1.62	2.92	11.51	1.17	1.50	0.1	10	42	0.87
7	M2	43.45	3.18	5.18	8.66	0.37	0.74	23.34	1.62	2.92	11.51	1.17	1.50	0.1	10	42	0.87
8	M2	43.33	3.17	5.00	8.52	0.36	0.73	23.34	1.62	2.92	11.51	1.17	1.33	0.1	10	42	0.87
9	M2	43.46	3.01	5.18	8.50	0.37	0.74	23.49	1.62	2.92	11.51	1.00	1.50	0.1	10	42	0.87
10	M3	43.61	3.18	5.18	8.64	0.37	0.92	23.49	1.62	2.92	11.51	1.17	1.33	0.1	60	49	1.52
11	M3	43.61	3.17	5.35	8.64	0.37	0.92	23.49	1.62	2.92	11.51	1.17	1.50	0.1	60	49	1.52
12	M3	43.97	3.19	5.37	8.79	0.37	0.93	23.69	1.62	2.92	11.52	1.17	1.50	0.1	60	49	1.52
13	M3	43.81	3.19	5.37	8.79	0.37	0.93	23.53	1.62	2.92	11.52	1.17	1.50	0.1	60	49	1.52
14	M3	43.81	3.36	5.04	8.79	0.56	0.75	23.53	1.62	2.92	11.53	1.17	1.34	0.1	60	49	1.52
15	M4	43.49	3.53	5.04	8.76	0.75	0.75	23.37	1.62	2.92	11.37	1.17	1.34	5	60	42	0.71
16	M4	43.66	3.36	5.04	8.79	0.56	0.75	23.53	1.62	2.92	11.37	1.17	1.34	5	60	42	0.71
17	M4	43.29	3.34	5.18	8.64	0.55	0.73	23.34	1.62	2.92	11.35	1.17	1.50	5	60	42	0.71
18	M4	43.13	3.34	5.18	8.64	0.55	0.73	23.18	1.62	2.92	11.35	1.17	1.50	5	60	42	0.71
19	M5	43.30	3.18	5.18	8.64	0.55	0.73	23.18	1.62	2.92	11.51	1.00	1.50	5	0	49	2.38
20	M5	43.13	3.34	5.18	8.64	0.55	0.73	23.02	1.62	2.92	11.51	1.17	1.50	5	0	49	2.38
21	M5	43.49	3.53	5.04	8.76	0.75	0.75	23.21	1.62	2.92	11.53	1.17	1.34	5	0	49	2.38
22	M5	43.49	3.53	5.04	8.76	0.75	0.75	23.21	1.62	2.92	11.53	1.17	1.34	5	0	49	2.38
23	M5	43.49	3.53	5.04	8.76	0.75	0.75	23.21	1.62	2.92	11.53	1.17	1.34	5	0	49	2.38
24	M5	43.32	3.53	5.21	8.76	0.75	0.75	23.06	1.62	2.92	11.52	1.17	1.50	5	0	49	2.38
25	M5	43.33	3.53	5.04	8.76	0.75	0.75	23.06	1.62	2.92	11.53	1.17	1.34	5	0	49	2.38
26	M5	43.32	3.53	5.21	8.76	0.75	0.75	23.06	1.62	2.92	11.52	1.17	1.50	5	0	49	2.38
27	M5	43.48	3.53	5.20	8.76	0.75	0.75	23.21	1.62	2.92	11.52	1.17	1.50	5	0	49	2.38
28	M5	44.89	3.64	5.03	8.91	0.76	0.76	24.04	2.02	2.86	11.96	0.87	1.39	5	0	49	2.38
29	M6	43.19	3.52	5.19	8.63	0.73	0.73	23.06	1.62	2.92	11.52	1.17	1.50	5	10	35	1.17
30	M6	43.19	3.52	5.19	8.63	0.73	0.73	23.06	1.62	2.92	11.52	1.17	1.50	5	10	35	1.17
31	M6	43.31	3.50	5.16	8.48	0.72	0.72	23.34	1.62	2.92	11.51	1.17	1.50	5	10	35	1.17
32	M6	43.16	3.33	5.17	8.48	0.72	0.72	23.18	1.62	2.92	11.51	1.00	1.50	5	10	35	1.17
33	M6	43.16	3.33	5.00	8.50	0.54	0.72	23.18	1.62	2.92	11.51	1.17	1.33	5	10	35	1.17
34	M7	43.15	3.33	5.33	8.48	0.54	0.90	23.18	1.62	2.92	11.51	1.17	1.50	10	10	49	3.79
35	M7	43.36	3.35	5.19	8.65	0.55	0.74	23.21	1.62	2.92	11.52	1.17	1.50	10	10	49	3.79
36	M7	43.20	3.35	5.19	8.65	0.55	0.74	23.06	1.62	2.92	11.52	1.17	1.50	10	10	49	3.79
37	M8	43.17	3.36	5.21	8.62	0.56	0.75	23.06	1.62	2.92	11.52	1.17	1.50	10	60	35	4.24
38	M8	42.88	3.35	5.19	8.49	0.55	0.74	22.90	1.62	2.92	11.52	1.17	1.50	10	60	35	4.24
39	M8	43.04	3.35	5.19	8.49	0.55	0.74	23.06	1.62	2.92	11.52	1.17	1.50	10	60	35	4.24
40	M8	43.36	3.35	5.19	8.65	0.55	0.74	23.21	1.62	2.92	11.52	1.17	1.50	10	60	35	4.24
41	M9	42.88	3.35	5.19	8.49	0.55	0.74	22.90	1.62	2.92	11.52	1.17	1.50	10	0	42	6.06
42	M9	43.01	3.36	5.21	8.49	0.55	0.74	23.03	1.63	2.94	11.52	1.17	1.50	10	0	42	6.06
43	M9	43.35	3.19	5.04	8.65	0.55	0.74	23.06	1.62	2.92	11.67	1.01	1.35	10	0	42	6.06
44	M9	42.88	3.52	5.03	8.65	0.55	0.74	22.90	1.62	2.92	11.36	1.34	1.34	10	0	42	6.06
Mean		43.40	3.34	5.15	8.65	0.55	0.76	23.27	1.63	2.92	11.51	1.15	14.11				2.29
SD		0.34	0.15	0.10	0.11	0.14	0.06	0.24	0.06	0.01	0.09	0.08	0.10				1.64

^1^ metAFLP characteristics: SV—sequence variation, DNMV—de novo methylation, DMV-demethylation; values of metAFLP quantitative characteristics expressed in percentages and evaluated for triticale anther culture-derived regenerants, CHH, CHG, CG—the sequence contexts in which the methylated cytosine may be located; M1–M9—trials with different in vitro conditions; GP—states for green regenerants obtained per 100 plated anthers; SD—standard deviation.

**Table 2:** Mediation analyses outcomes depicting relationships between DNMV (CHH_DNMV) and SV (CHH_SV) mediated by copper ions, CHH_SV and CHH_DNMV and GP with copper ions as a mediator and CG_DNMV and GP mediated by CG_SV. Both unstandardized (B) and standardized (*β*) coefficients are presented.

Simple Mediation (IV → M → DV) ^1^	Statistics			
	Path a	Path b and c’	Path c	Indirect Effect
DNMV → Cu → SV	*F*(1,42) = 17.4334, *MSE* = 11.4947, *p* = 0.0001, *R*^2^ = 0.2046:	*F*(2.41) = 15.8139, *MSE* = 0.0803, *p* < 0.0001, *R*^2^ = 0.3389	*F*(1.42) = 0.0246, *MSE* = 0.1183, *p* = 0.8762, *R*^2^ = 0.0015	B = −0.6771, *se* = 0.2239, CI[−1.1570;−0.2897]
	(a) B = 11.4812, *se* = 2.7498, *t*(42) = 4.1753, *p* = 0.0001, 95%CI[5.9319;17.0305], *β* = 0.4523	(b) B = −0.0590, *se* = 0.0144, *t*(41) = −4.0885, *p* = 0.0002, 95%CI[−0.0881;−0.0298], *β* = −0.6512	(c) B = −0.0899, *se* = 0.5736, *t*(42) = −0.1567, *p* = 0.8762, 95%CI[−1.2474;1.0676], *β* = −0.0391	
		(c’) B = 0.5872, *se* = 0.6351, *t*(41) = 0.9245, *p* = 0.3606, 95%CI[−0.6955;1.8699], β = 0.2554		
CHH_DNMV → Cu → CHH_SV	*F*(1,42) = 26.874, *MSE* = 10.985, *p* < 0.0001, *R*^2^ = 0.2398	*F*(2.41) = 7.7133, *MSE* = 0.0087, *p* = 0.0014, *R*^2^ = 0.2837	*F*(1.42) = 2.4947, *MSE* = 0.0111, *p* = 0.1217, *R*^2^ = 0.0590	B = −0.1966, *se* = 0.0637, CI[−0.3311;−0.0792]
	(a) B = 12.6377, *se* = 2.4378, *t*(42) = 5.184, *p* < 0.0001, 95%CI[7 0.7179;17.5575], *β* = 0.4897	(b) B = −0.0156, *se* = 0.0041, *t*(41) = −3.7754, *p* = 0.0005, 95%CI[−0.0239;−0.0072], *β* = −0.5438	(c) B = 0.1792, *se* = 0.1135, *t*(42) = 1.5795, *p* = 0.1217, 95%CI[−0.0498;0.4083], *β* = 0.2428	
		(c’) B = 0.3758, *se* = 0.1251, *t*(41) = 3.0038, *p* = 0.0045, 95%CI[0.1231;0.628], *β* = 0.5091		
CHH_DNMV → Cu → GP	*F*(1,42) = 26.8740, *MSE* = 10.9850, *p* < 0.0001, *R*^2^ = 0.2398	*F*(2.41) = 54.9096, *MSE* = 0.7443, *p* < 0.0001, *R*^2^ = 0.7363	*F*(1.42) = 3.9786, *MSE* = 2.6641, *p* = 0.0526, *R*^2^ = 0.0332	B = 5.3075, *se* = 1.2321, CI[3.0808;8.0131]
	(a) B = 12.6377, *se* = 2.4378, *t*(42) = 5.1840, *p* < 0.0001, 95%CI[7.7179;17.5575], *β* = 0.4897	(b) B = 0.4200, *se* = 0.0468, *t*(41) = 8.9768, *p* < 0.0001, 95%CI[0.3255;0.5145], *β* = 0.9618	(c) B = 2.0519, *se* = 1.0287, *t*(42) = 1.9946, *p* = 0.0526, 95%CI[−0.0241;4.127], *β* = 0.1821	
		(c’) B = −3.2556, *se* = 0.9286, *t*(41) = −3.5060, *p* = 0.0011, 95%CI[−5.1309;−1.3803], *β* = −0.2889		
CHH_SV → Cu → GP	*F*(1,42) = 4.5538, *MSE* = 13.1980, *p* = 0.0387, *R*^2^ = 0.0867	*F*(2.41) = 42.4471, *MSE* = 0.9234, *p* < 0.0001, *R*^2^ = 0.6729	*F*(1.42) = 2.3942, *MSE* = 2.5955, *p* = 0.1293, *R*^2^ = 0.0581	B = −3.6875, *se* = 1.7244, CI[−7.0939;−0.4042]
	(a) B = −10.2925, *se* = 4.8232, *t*(42) = −2.1340, *p* = 0.0387, 95%CI[−20.0261;−0.5588], *β* = −0.2944	(b) B = 0.3583, *se* = 0.0389, *t*(41) = 9.2089, *p* < 0.0001, 95%CI[0.2797;0.4368], *β* = 0.8205	(c) B = −3.6780, *se* = 2.3770, *t*(42) = −1.5473, *p* = 0.1293, 95%CI[−8.4752;1.1191], *β* = −0.2410	
		(c’) B = 0.0094, *se* = 1.4308, *t*(41) = 0.0066, *p* = 0.9948, 95%CI[−2.8802;2.8990], *β* = 0.0006		
CHG_SV → Cu → GP	*F*(1,42) = 7.1830, *MSE* = 7.5428, *p* = 0.0105, *R*^2^ = 0.6914	*F*(2.41) = 43.5602, *MSE* = 0.9196, *p* < 0.0001, *R*^2^ = 0.6742	*F*(1.42) = 6.1337, *MSE* = 1.7838, *p* = 0.0174, *R*^2^ = 0.3526	B = −3.7818, *se* = 1.1871, CI[−6.5858;−2.0545]
	(a) B = −11.0336, *se* = 4.1169, *t*(42) = −2.6801, *p* = 0.0105, 95%CI[−19.3419;−2.7254], *β* = −0.6914	(b) B = 0.3428, *se* = 0.0432, *t*(41) = 7.9321, *p* < 0.0001, 95%CI[0.2555;0.4300], *β* = 0.7849	(c) B = −4.1381, *se* = 1.67090, *t*(42) = −2.4766, *p* = 0.0174, 95%CI[−7.5100;−0.7661], *β* = 0.5938	
		(c’) B = −0.3563, *se* = 0.7581, *t*(41) = −0.4700, *p* = 0.6409, 95%CI[−1.8874;1.1748], *β* = −0.0511		
CG_DNMV → CG_SV → GP	*F*(1,42) = 4.5366, *MSE* = 0.0045, *p* = 0.0391, *R*^2^ = 0.4927	*F*(2.41) = 3.3356, *MSE* = 2.5015, *p* = 0.0455, *R*^2^ = 0.1138	*F*(1.42) = 0.0336, *MSE* = 2.7514, *p* = 0.8554, *R*^2^ = 0.0015	B = −7.1420, *se* = 3.3000, CI[−13.8242;−1.1299]
	(a) B = −0.8583, *se* = 0.4030, *t*(42) = −2.1299, *p* = 0.0391, 95%CI[−1.6716;−0.0451 ], *β* = −0.7019	(b) B = 8.3208, *se* = 4.0779, *t*(41) = 2.0405, *p* = 0.0478, 95%CI[0.0853;16.5564], *β* = 0.4705	(c) B = 0.8307, *se* = 4.5290, *t*(42) = 0.1834, *p* = 0.8554, 95%CI[−8.3093;9.9707], *β* = 0.0384	
		(c’) B = 7.9727, *se* = 4.7204, *t*(41) = 1.6890, *p* = 0.0988, 95%CI[−1.5605;17.5058], *β* = 0.3687		

^1^ IV—independent variables; M—mediator; DV—dependent variable; The DNMV, SV, CHH_SV, CHH_DNMV, CHG_DNMV, and CG_SV are the metAFLP quantitative characteristics expressed in percentages and evaluated for triticale anther culture-derived regenerants and observed in specific sequences (CHH, CHG, CG). The GP states for green regenerants; a = effect of the predictor (IV) on the mediator (M); b = effect of the mediator on the outcome (DV); c = total effect of focal predictor (IV) on the outcome (DV); c’ = direct effect of predictor (IV) on the outcome (DV) while controlling for the mediator; IE = indirect effect of predictor (IV) on the outcome (DV) through the mediator (M); R2 = the amount of variance explained by the model; SE = standard error; *p* = probability; 95%LLCI and ULCI = 95% lower and upper confidence interval; IE = indirect effect; CI (under IE) bootstrap values of confidence interval (5000 bootstraps).

## Data Availability

Not applicable.

## Supplementary Materials

The following are available online at https://www.mdpi.com/article/10.3390/cells11010084/s1, Table S1: Oligonucleotides applied for metAFLP in triticale.

## Abbreviations

2,4-D	2,4-Dichlorophenoxyacetic Acid
AOX	Alternative Oxidase
COX	Cytochrome *c* Oxidase
DMV	Demethylation
DNMT	DNA Methyltransferase
DNMV	De Novo Methylation
ETC	Electron Transport Chain
GP	Green Plants
GSH	Glutathione
IAA	Indole-3-Acetic Acid
IM	Induction Medium
metAFLP	Methylatio-Sensitive Amplified Fragment Length Polymorphism
ROS	Reactive Oxygen Species
SAM	S-Adenosyl-L-Methionine
SV	Sequence Variation
TCIV	Tissue Culture-Induced Variation

## References

[1] Orłowska R., Pachota K.A., Machczyńska J., Niedziela A., Makowska K., Zimny J., Bednarek P.T. (2020). Improvement of anther cultures conditions using the Taguchi method in three cereal crops. Electron. J. Biotechnol..

[2] Warchoł M., Juzoń K., Dziurka K., Czyczyło-Mysza I., Kapłoniak K., Marcińska I., Skrzypek E. (2021). The effect of zinc, copper, and silver ions on oat (*Avena sativa* L.) androgenesis. Plants.

[3] Sharma S.S., Dietz K.-J. (2009). The relationship between metal toxicity and cellular redox imbalance. Trends Plant Sci..

[4] Pilon M., Cohu C.M., Ravet K., Abdel-Ghany S.E., Gaymard F. (2009). Essential transition metal homeostasis in plants. Curr. Opin. Plant Biol..

[5] Lee C.-Y., Chang C.-L., Wang Y.-N., Fu L.-M. (2011). Microfluidic Mixing: A Review. Int. J. Mol. Sci..

[6] Vanlerberghe G.C. (2013). Alternative oxidase: A mitochondrial respiratory pathway to maintain metabolic and signaling homeostasis during abiotic and biotic stress in plants. Int. J. Mol. Sci..

[7] Moller I.M. (2001). Plant mitochondria and oxidative stress: Electron transport, nadph turnover, and metabolism of reactive oxygen species. Annu. Rev. Plant Physiol. Plant Mol. Biol..

[8] Garcia L., Welchen E., Gonzalez D.H. (2014). Mitochondria and copper homeostasis in plants. Mitochondrion.

[9] Halliwell B. (2006). Reactive species and antioxidants. Redox biology is a fundamental theme of aerobic life. Plant Physiol..

[10] Manceau A., Simionovici A., Lanson M., Perrin J., Tucoulou R., Bohic S., Fakra S.C., Marcus M.A., Bedell J.P., Nagy K.L. (2013). Thlaspi arvense binds Cu(II) as a bis-(l-histidinato) complex on root cell walls in an urban ecosystem. Metallomics.

[11] Gizatullin A., Becker J., Islamov D., Serov N., Schindler S., Klimovitskii A., Shtyrlin V. (2021). Synthesis and structure of a complex of copper(I) with l-cysteine and chloride ions containing Cu12S6 nanoclusters. Acta Crystallographica Section E.

[12] Burkhead J.L., Gogolin Reynolds K.A., Abdel-Ghany S.E., Cohu C.M., Pilon M. (2009). Copper homeostasis. New Phytol..

[13] Mäkinen K., De S. (2019). The significance of methionine cycle enzymes in plant virus infections. Curr. Opin. Plant Biol..

[14] Roje S. (2006). S-Adenosyl-L-methionine: Beyond the universal methyl group donor. Phytochemistry.

[15] Chen Y., Zou T., McCormick S. (2016). S-Adenosylmethionine synthetase 3 is important for pollen tube growth. Plant Physiol..

[16] Heidari P., Mazloomi F., Nussbaumer T., Barcaccia G. (2020). Insights into the SAM synthetase gene family and its roles in tomato seedlings under abiotic stresses and hormone treatments. Plants.

[17] Hasanuzzaman M., Nahar K., Anee T.I., Fujita M. (2017). Glutathione in plants: Biosynthesis and physiological role in environmental stress tolerance. Physiol. Mol. Biol. Plants Int. J. Funct. Plant Biol..

[18] Giovanelli J., Mudd S.H., Datko A.H. (1978). Homocysteine biosynthesis in green plants. Physiological importance of the transsulfuration pathway in *Chlorella sorokiniana* growing under steady state conditions with limiting sulfate. J. Biol. Chem..

[19] Meng J., Wang L., Wang J., Zhao X., Cheng J., Yu W., Jin D., Li Q., Gong Z. (2018). Methionine adenosyltransferase4 mediates DNA and histone methylation. Plant Physiol..

[20] Solís M.-T., Rodríguez-Serrano M., Meijón M., Cañal M.-J., Cifuentes A., Risueño M.C., Testillano P.S. (2012). DNA methylation dynamics and MET1a-like gene expression changes during stress-induced pollen reprogramming to embryogenesis. J. Exp. Bot..

[21] He X.-J., Chen T., Zhu J.-K. (2011). Regulation and function of DNA methylation in plants and animals. Cell Res..

[22] Kumar S., Chinnusamy V., Mohapatra T. (2018). Epigenetics of modified DNA bases: 5-methylcytosine and beyond. Front. Genet..

[23] Lee D.H., O’Connor T.R., Pfeifer G.P. (2002). Oxidative DNA damage induced by copper and hydrogen peroxide promotes CG→TT tandem mutations at methylated CpG dinucleotides in nucleotide excision repair-deficient cells. Nucleic Acids Res..

[24] Kusmartsev V., Drożdż M., Schuster-Böckler B., Warnecke T. (2020). Cytosine methylation affects the mutability of neighboring nucleotides in germline and soma. Genetics.

[25] Orłowska R., Zimny J., Bednarek P.T. (2021). Copper ions induce DNA sequence variation in zygotic embryo culture-derived barley regenerants. Front. Plant Sci..

[26] Bednarek P.T., Zebrowski J., Orłowska R. (2020). Exploring the biochemical origin of DNA sequence variation in barley plants regenerated via in vitro anther culture. Int. J. Mol. Sci..

[27] Bednarek P.T., Orłowska R. (2020). Time of in vitro anther culture may moderate action of copper and silver ions that affect the relationship between DNA methylation change and the yield of barley green regenerants. Plants.

[28] Ciriolo M.R., Civitareale P., Carrì M.T., De Martino A., Galiazzo F., Rotilio G. (1994). Purification and characterization of Ag,Zn-superoxide dismutase from Saccharomyces cerevisiae exposed to silver. J. Biol. Chem..

[29] Grozeva S., Nankar A.N. (2020). Effect of incubation period and culture medium on pepper anther culture. Indian J. Biotechnol..

[30] Sato S., Katoh N., Iwai S., Hagimori M. (2002). Effect of low temperature pretreatment of buds or inflorescence on isolated microspore culture in *Brassica rapa* (syn. B. campestris). Breed. Sci..

[31] Mikuła A., Tomiczak K., Rybczynski J.J. (2011). Cryopreservation enhances embryogenic capacity of *Gentiana cruciata* L. suspension culture and maintains (epi)genetic uniformity of regenerants. Plant Cell Rep..

[32] Machczyńska J., Orłowska R., Zimny J., Bednarek P.T. (2014). Extended metAFLP approach in studies of the tissue culture induced variation (TCIV) in case of triticale. Mol. Breed..

[33] Orłowska R., Bednarek P.T. (2020). Precise evaluation of tissue culture-induced variation during optimisation of in vitro regeneration regime in barley. Plant Mol. Biol..

[34] Fiuk A., Bednarek P.T., Rybczyński J.J. (2010). Flow cytometry, HPLC-RP, and metAFLP analyses to assess genetic variability in somatic embryo-derived plantlets of *Gentiana pannonica* Scop. Plant Mol. Biol. Report..

[35] Śliwińska A.A., Białek A., Orłowska R., Mańkowski D., Sykłowska-Baranek K., Pietrosiuk A. (2021). Comparative study of the genetic and biochemical variability of *Polyscias filicifolia* (Araliaceae) regenerants obtained by indirect and direct somatic embryogenesis as a source of triterpenes. Int. J. Mol. Sci..

[36] Hayes A.F. (2018). Introduction to Mediation, Moderation, and Conditional Process Analysis. A Regression Bases Approach.

[37] Bednarek P.T., Orłowska R., Mańkowski D.R., Oleszczuk S., Zebrowski J. (2021). Structural Equation Modeling (SEM) analysis of sequence variation and green plant regeneration via anther culture in barley. Cells.

[38] Zhuang J.J., Xu J., Hu H., Vega M.R. (1983). Increasing differentiation frequencies in wheat pollen callus. Cell and Tissue Culture Techniques for Cereal Crop Improvement.

[39] Chu C.C., Hu H. (1978). The N6 medium and its applications to anther culture of cereal crops. Proc. Symp. Plant Tissue Culture.

[40] Bednarek P.T., Orłowska R., Koebner R.M.D., Zimny J. (2007). Quantification of the tissue-culture induced variation in barley (*Hordeum vulgare* L.). BMC Plant Biol..

[41] Faul F., Erdfelder E., Buchner A., Lang A.-G. (2009). Statistical power analyses using G*Power 3.1: Tests for correlation and regression analyses. Behav. Res. Methods.

[42] Addinsoft (2020). XLSTAT Statistical and Data Analysis Solution. https://www.xlstat.com.

[43] Yang S.F., Hoffman N.E. (1984). Ethylene biosynthesis and its regulation in higher plants. Annu. Rev. Plant Physiol..

[44] Bednarek P.T., Orłowska R. (2020). CG demethylation leads to sequence mutations in an anther culture of barley due to the presence of Cu, Ag ions in the medium and culture time. Int. J. Mol. Sci..

[45] Festa R.A., Thiele D.J. (2011). Copper: An essential metal in biology. Curr. Biol..

[46] Cong W., Miao Y., Xu L., Zhang Y., Yuan C., Wang J., Zhuang T., Lin X., Jiang L., Wang N. (2019). Transgenerational memory of gene expression changes induced by heavy metal stress in rice (*Oryza sativa* L.). BMC Plant Biol..

[47] Law J.A., Jacobsen S.E. (2010). Establishing, maintaining and modifying DNA methylation patterns in plants and animals. Nat. Rev. Genet..

[48] Kankel M.W., Ramsey D.E., Stokes T.L., Flowers S.K., Haag J.R., Jeddeloh J.A., Riddle N.C., Verbsky M.L., Richards E.J. (2003). Arabidopsis MET1 cytosine methyltransferase mutants. Genetics.

[49] Bray C.M., West C.E. (2005). DNA repair mechanisms in plants: Crucial sensors and effectors for the maintenance of genome integrity. New Phytol..

[50] Machczyńska J., Orłowska R., Mańkowski D.R., Zimny J., Bednarek P.T. (2014). DNA methylation changes in triticale due to in vitro culture plant regeneration and consecutive reproduction. Plant Cell Tissue Organ Cult..

[51] Orłowska R., Machczyńska J., Oleszczuk S., Zimny J., Bednarek P.T. (2016). DNA methylation changes and TE activity induced in tissue cultures of barley (*Hordeum vulgare* L.). J. Biol. Res..

[52] Valentine J.S., de Freitas D.M. (1985). Copper-zinc superoxide dismutase: A unique biological “ligand” for bioinorganic studies. J. Chem. Educ..

[53] Roe J.A., Peoples R., Scholler D.M., Valentine J.S. (1990). Silver-binding properties of bovine cuprozinc superoxide dismutase and the overall stability of selected metalion derivatives. J. Am. Chem. Soc..

[54] Çekiç F.O., Ekinci S., Inal M., Ozakca D. (2017). Silver nanoparticles induced genotoxicity and oxidative stress in tomato plants. Turk. J. Biol..

[55] Bairu M.W., Fennell C.W., van Staden J. (2006). The effect of plant growth regulators on somaclonal variation in Cavendish banana (*Musa* AAA cv. ‘*Zelig*’). Sci. Hortic..

[56] Matzke M.A., Mosher R.A. (2014). RNA-directed DNA methylation: An epigenetic pathway of increasing complexity. Nat. Rev. Genet..

[57] Schmitz R.J., Schultz M.D., Lewsey M.G., O’Malley R.C., Urich M.A., Libiger O., Schork N.J., Ecker J.R. (2011). Transgenerational epigenetic instability is a source of novel methylation variants. Science.

[58] Becker C., Hagmann J., Müller J., Koenig D., Stegle O., Borgwardt K., Weigel D. (2011). Spontaneous epigenetic variation in the Arabidopsis thaliana methylome. Nature.

[59] Grin I., Ishchenko A.A. (2016). An interplay of the base excision repair and mismatch repair pathways in active DNA demethylation. Nucleic Acids Res..

[60] Drohat A.C., Coey C.T. (2016). Role of base excision “repair” enzymes in erasing epigenetic marks from DNA. Chem. Rev..

[61] Klungland A., Robertson A.B. (2017). Oxidized C5-methyl cytosine bases in DNA: 5-Hydroxymethylcytosine; 5-formylcytosine; and 5-carboxycytosine. Free Radic. Biol. Med..

[62] Tang Y., Xiong J., Jiang H.-P., Zheng S.-J., Feng Y.-Q., Yuan B.-F. (2014). Determination of Oxidation Products of 5-Methylcytosine in Plants by Chemical Derivatization Coupled with Liquid Chromatography/Tandem Mass Spectrometry Analysis. Anal. Chem..

[63] Ji L., Jordan W.T., Shi X., Hu L., He C., Schmitz R.J. (2018). TET-mediated epimutagenesis of the Arabidopsis thaliana methylome. Nat. Commun..

[64] Shi D.Q., Ali I., Tang J., Yang W.C. (2017). New Insights into 5hmC DNA Modification: Generation, Distribution and Function. Front. Genet..

[65] Domb K., Katz A., Yaari R., Kaisler E., Nguyen V.H., Hong U.V.T., Griess O., Heskiau K.G., Ohad N., Zemach A. (2020). Non-CG methylation is superior to CG methylation in genome regulation. bioRxiv.

[66] Zemach A., Kim M.Y., Silva P., Rodrigues J.A., Dotson B., Brooks M.D., Zilberman D. (2010). Local DNA hypomethylation activates genes in rice endosperm. Proc. Natl. Acad. Sci. USA.

[67] Zemach A., McDaniel I.E., Silva P., Zilberman D. (2010). Genome-wide evolutionary analysis of eukaryotic DNA methylation. Science.

[68] Park K., Kim M.Y., Vickers M., Park J.-S., Hyun Y., Okamoto T., Zilberman D., Fischer R.L., Feng X., Choi Y. (2016). DNA demethylation is initiated in the central cells of *Arabidopsis* and rice. Proc. Natl. Acad. Sci. USA.

[69] Nebert D.W., Vasiliou V. (2004). Analysis of the glutathione S-transferase (GST) gene family. Hum. Genom..

