# Supplementary material for: Triticale Green Plant Regeneration Is Due to DNA Methylation and Sequence Changes Affecting Distinct Sequence Contexts in the Presence of Copper Ions in Induction Medium
Source: Cells. 2021 Dec 28;11(1):84. doi: 10.3390/cells11010084 (PMC8750698; doi:10.3390/cells11010084)
Supplement: Supplementary file 1 [file cells-11-00084-s001.zip › cells-1477272-supplementary.pdf]

Table S1 Oligonucleotides applied for metAFLP in triticale

metAFLP oligomer	Sequence 5'→3'
Adaptors	
Ad1 *Acc65*I	CTC GTA GCA TGC GTA CA
Ad2 *Acc65*I	GTA CTGTACGCATGCTAC
Ad1 *KpnI*	CTC GTA GCA TGC GTA CAG TAC
Ad2 *KpnI*	TGTACGCATGCTAC
Ad1 *MseI*	TAC TCA GGA CTC ATC
Ad2 *MseI*	GAC GAT GAG TCC TGA G
Preselective primers	GAT GAG TCC TGA GTA AC
Presel Acc56I/*KpnI*	GCA TGC GTA CAG TAC C
Presel *MseI*	GAT GAG TCC TGA GTA AC
Labeled ^{32}P selective oligonucleotides	
CG-GAC	CA TGC GTA CAG TAC CGA C
CG-GCA	CA TGC GTA CAG TAC CGC A
CG-GGC	CA TGC GTA CAG TAC CGG C
CG-TCG	CA TGC GTA CAG TAC CTC G
CXG-AGA	CA TGC GTA CAG TAC CAG A
CXG-AGC	CA TGC GTA CAG TAC CAG C
CXG-AGG	CA TGC GTA CAG TAC CAG G
CXG-ATG	CA TGC GTA CAG TAC CAT G
CXG-TGC	CA TGC GTA CAG TAC CTG C
CXG-TTG	CA TGC GTA CAG TAC CTT G
CXX-ATT	CA TGC GTA CAG TAC CAT T
CXX-TAA	CA TGC GTA CAG TAC CTA A
Selective oligonucleotides	
M-CAC	GAT GAG TCC TGA GTA ACA C
M-CGT	GAT GAG TCC TGA GTA ACG T
